# Metagenomics and untargeted metabolomics reveal antibiotic resistance dynamics in an anaerobic digestion–composting system treating organic fraction of municipal solid waste

**DOI:** 10.1186/s40793-025-00769-4

**Published:** 2025-08-14

**Authors:** Elisabetta Fanfoni, Paolo Bellassi, Alessandra Fontana, Erika Sinisgalli, Gabriele Rocchetti, Sergio Piccinini, Lorenzo Morelli, Fabrizio Cappa

**Affiliations:** 1 Department for Sustainable Food Process (DiSTAS), Università Cattolica del Sacro Cuore, Cremona, Italy; 2 Centro Ricerche Produzioni Animali, CRPA Soc. Cons. p. A, Reggio Emilia, Italy; 3 Department of Animal Science, Food and Nutrition (DiANA), Università Cattolica del Sacro Cuore, Cremona, Italy

**Keywords:** Waste treatment, Anaerobic fermentation, Aerobic composting, Shotgun sequencing, Resistome, Antibiotic resistance gene, Antibiotic compound, Digestate, Compost

## Abstract

**Background:**

The growing population and associated increase in municipal solid waste (MSW) have promoted the use of sustainable waste management strategies. Given its high organic content, MSW can be treated through anaerobic digestion (AD) and aerobic composting (AC) to recover value-added products such as bioenergy and soil amendments. However, MSW is also recognized as a relevant source of antibiotic resistance genes (ARGs), raising concerns about environmental and public health impacts. This study aimed to elucidate the dynamics of ARGs and antibiotic compounds during the treatment of the organic fraction of municipal solid waste (OFMSW) through an integrated AD–AC system. By combining metagenomics and untargeted metabolomics, a comprehensive characterization of shifts in the microbial community, ARGs, and antibiotic compounds throughout the treatment stages was achieved. Shotgun sequencing enabled an in-depth resistome analysis based on metagenome-assembled genomes (MAGs), while untargeted metabolomics revealed the occurrence and transformation of antibiotic compounds across the system.

**Results:**

The integrated process resulted in a significant differentiation of microbial communities, resistome, and antibiotic compounds profiles, at different stages of the waste treatment plant. AD samples were mostly dominated by aminoglycoside and lincosamide ARGs, whereas AC samples by macrolide and rifamycin ARGs. Despite differences in drug class dominance, the composting process significantly increased both the ARGs diversity (i.e., digestate: H = 2.6 ± 0.1; mature compost: H = 3.7 ± 0.1) and abundance (i.e., mature compost vs. digestate: log2(FC) = 3.7). Untargeted metabolomics revealed distinct distributions of antibiotics among the six matrices (i.e., pulp, digestate, solid fraction, liquid fraction, fresh compost, and mature compost) suggesting limited degradation or transformation of some classes during treatment. Digestate was enriched in phenazines and trimethoprim derivatives, whereas mature compost mainly included phenicols and sulfonamides.

**Conclusions:**

This study provides valuable insights into the fate of antibiotic resistance genes and the persistence of antibiotic compounds in an integrated AD and AC system treating OFMSW. Moreover, it was shown how the integration of -omics techniques as metagenomics and metabolomics can be systematically utilized to detect emerging ARGs and antibiotic compounds dynamics and monitor their ongoing evolution in biological waste treatment plants.

**Supplementary Information:**

The online version contains supplementary material available at 10.1186/s40793-025-00769-4.

## Background

The expansion of urbanization and population growth naturally lead to an increase in municipal solid waste (MSW). MSW contains approximately 40–70% organic fractions (OF), and food is the major component [1] making them suitable for both anaerobic digestion (AD) and aerobic composting (AC) treatments. These two biological-based procedures can be also exploited in an integrated process, producing biogas, thus renewable energy, and compost, which is largely used in agriculture as amendment to support and integrate the organic fraction in soil. In fact, in anaerobic conditions, the digestion of different organic substrates is carried out by symbiotic microorganisms, which transform organic materials into biogas, and digestate as by-product [2]. On the other side, AC is a biological process carried out by microorganisms in aerobic conditions that can improve the quality of the digestate resulted from AD, in terms of nutrients and stability [3]. The final compostable substances can be then exploited in the agronomic field and horticulture industries. However, to guarantee environmental safety, it is relevant to have a deep knowledge of the microorganisms and relative genes of concern included in the digestate and compost for their field application as fertilizers. In fact, the agronomic use of composted organic waste could introduce microorganisms carrying antibiotic resistance genes (ARGs) into the soil, with the associated risk of horizontal gene transfer to different microbial species [4], as well as recirculation in the food chain, posing a risk to human health [5]. Indeed, OFMSW is usually composed of material resulting from the separate collection of organic wastes (food residues or food preparations and assimilable fractions, such as food paper soiled with food residues). Those wastes are considered a significant source of ARGs [6], due to the massive and inappropriate use of antibiotics by humans, but also in the agriculture and livestock farming context [7]. The interest in considering AD and AC for the disposal of organic fraction of municipal solid waste (OFMSW) can also be their exploitation to contrast the spread of ARGs. Through these two biological processes, microbial communities transition from the initial substrate to the final products (digestate and compost), leading to a shift in the overall predicted genetic content (i.e., microbiome), including the ARG profile (i.e., resistome). This transformation can be thoroughly examined using a metagenomic approach [8, 9]. Indeed, metagenomics serves as a powerful tool for detecting emerging ARGs and monitoring their ongoing evolution [10].

A recent review has evaluated in detail the effectiveness of organic waste treatment plants in reducing ARGs, particularly in anaerobic digestion and composting systems. Anaerobic digestion has shown variable effectiveness in reducing ARGs; while some studies showed a decrease in specific classes (tetracyclines, sulfonamides, macrolides, fluoroquinolones, trimethoprim, beta-lactamase, aminoglycosides, florfenicol) [11], others reported a persistence or even enrichment, especially under mesophilic conditions [8]. Composting, on the other hand, seemed to be more effective, thanks to the high temperatures reached in the thermophilic phase, which contribute to the degradation of extracellular DNA and the reduction of ARG-carrying bacteria. However, effectiveness depends heavily on the duration and management of the process [12, 13]. Integrated systems of anaerobic digestion followed by composting appear to offer more promising results, combining anaerobic organic stabilization with the thermal and oxidative effect of composting, achieving a more marked and consistent reduction in ARGs [14, 15].

Considering the fate of antibiotic chemical compounds in anaerobic digestion and composting of different organic waste treatments, targeted methods have been mostly applied, such as liquid chromatography with tandem mass spectrometry (LC-MS/MS), to evaluate the presence of specific class of compounds. Anaerobic digestion removal rates depend on the antibiotic type, process parameters such as temperatures (thermophilic vs. mesophilic), retention time, digester design, and the use of additives like zero-valent iron [16–20]. In contrast, composting offers higher degradation potential due to thermophilic temperatures, aerobic microbial activity, and oxidative processes, especially for fluoroquinolones and macrolides, while sulfonamides were more persistent [15].

Several recent studies have adopted integrated strategies that combine metagenomic profiling with targeted antibiotic analysis to investigate microbial dynamics, ARGs content and antibiotic compounds fate in different organic waste treatment processes [11, 21, 22]. However, to the best of our knowledge, these investigations have not considered the OFMSW treatment or an integrated AD-AC full-scale process [15]. Moreover, the use of targeted methods implies to focus on a limited number of antibiotics, which, while sensitive and accurate, do not capture the full range of compounds present in the treated organic waste. An untargeted metabolomics approach can be employed to assess the presence of antibiotic compounds in compost and digestate samples. In recent years, this technique has gained increasing attention due to its ability to detect metabolites produced by microorganisms, enabling not only a comprehensive overview of the biological system under investigation but also precise assessments tailored to specific areas of interest [23].

To date, no studies have reported the combined application of metagenomic and untargeted metabolomic approaches in full-scale AD and AC systems, particularly with a focus on the resistome and antibiotic compound profiles. This study addresses this gap by investigating a full-scale integrated AD–AC treatment plant processing OFMSW, with the aim of assessing the dynamics of the microbial communities and the related resistome involved in the process, along with the antibiotic compounds profiling throughout the treatment chain. DNA shotgun sequencing enabled an in-depth resistome-based analysis of the metagenome-assembled genomes (MAGs) consortia under investigation, whereas untargeted metabolomic analysis gained deeper insights into the dynamics of antibiotic compounds.

## Methods

### Biowaste treatment plant characteristics

**Fig. 1:**
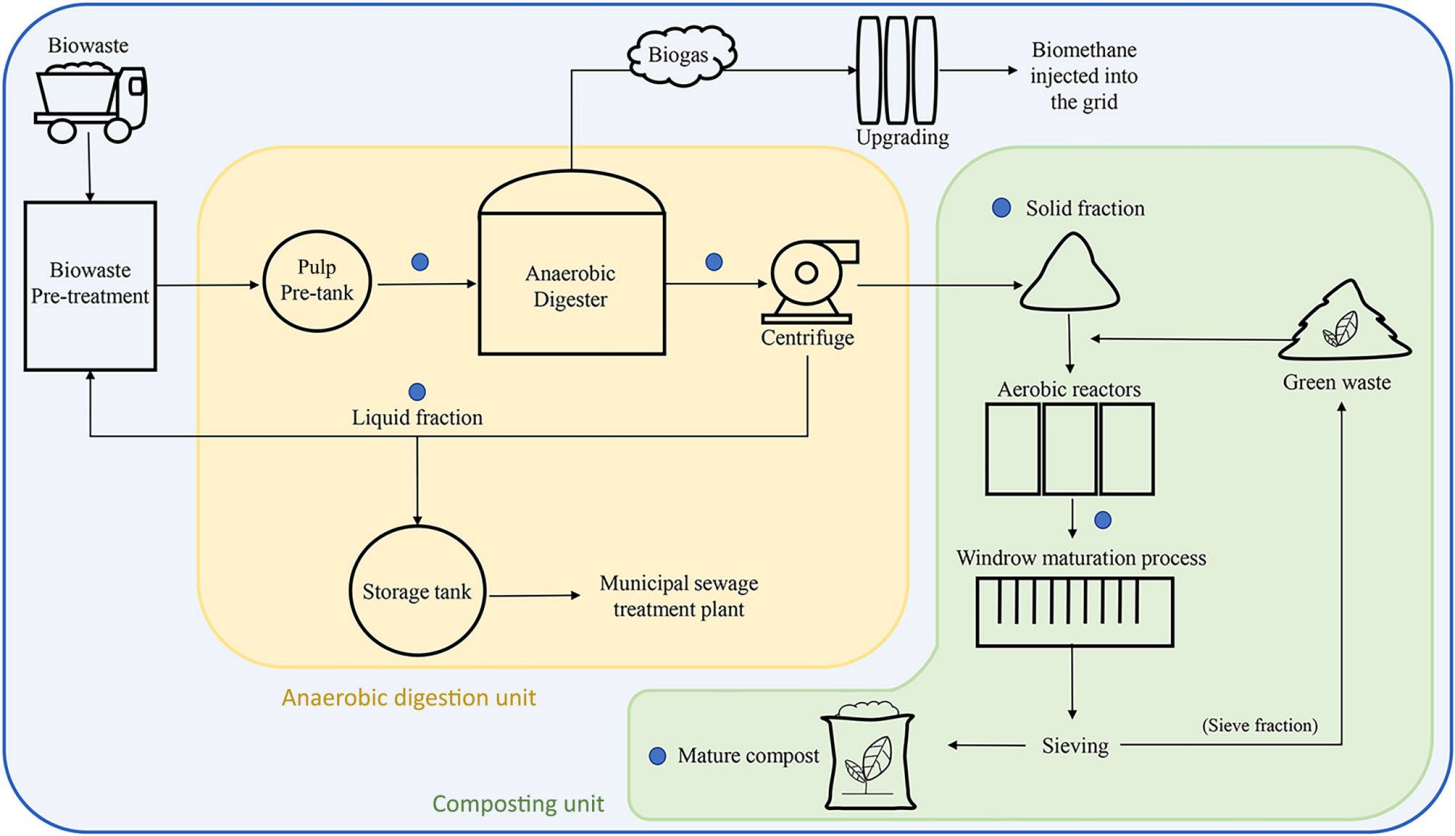
Graphical representation of the OFMSW treatment plant. The six sampling points investigated in this study are highlighted (blue dots)

The biomethane plant object of this study (Fig. 1) was an integrated anaerobic digestion and composting plant fed with OFMSW (biowaste) and situated in Northern Italy. The plant included a pre-treatment section of incoming waste, followed by an anaerobic digestion section operating in mesophilic regime (42 ± 2 °C) to produce biomethane through a membrane biogas upgrading system, and an aerobic stabilization section aimed at compost production. The pre-treatment section was aimed to remove inert materials (i.e., plastic, metals, and sand) prior to squeeze the remaining organic fraction to obtain a homogeneous pulp suitable for anaerobic digestion process. The AD was conducted in a wet regime, with a total solids (TS) concentration lower than 10%, an organic loading rate (OLR), calculated based on volatile solids (VS), of 2.06 kg VS/m^3^/day, and hydraulic retention time (HRT) of 23 days. The outcoming digestate underwent centrifugal separation: the liquid fraction was recirculated and used to form the pulp, while the solid fraction, together with green waste, was addressed to aerobic stabilization to produce mature compost. Specifically, a first stabilization phase was carried out in biocell units for 20 days at 60 ± 5 °C, then a second phase was performed in windrows for curing for 30 days, where temperatures gradually decline from 42 ± 3°C to ambient temperature. Compost stability was assessed through the Oxygen Uptake Rate test (UNI EN 16087-1:2020), yielding a mean value of 4.20 ± 0.97 mg O_2_/g VS·h, and confirming the completion of compost maturation.

### Sampling procedures and physico-chemical parameters

The plant was sampled once a month during May, June, and July 2021, considering each month as a biological replicate, and collecting one sample from six different sites, for a total of 18 samples (i.e., three replicate for each matrix): pulp, digestate, solid fraction, liquid fraction, fresh compost, and mature compost (Fig. 1). The sampling valves and the equipment used to collect digestate samples were previously cleaned with 0.05% sodium hypochlorite solution to limit exogenous bacterial contamination. About 350 mL of each sample type was transferred in sterile bottles (500 mL; LP Italiana, Milan, Italy) and cooled at 4 °C to prevent further biological changes. The main physico-chemical parameters were measured according to standard procedures [24] and were monitored as follows: TS and VS were measured for all sample categories (Table S1); pH, acidity/alkalinity ratio (FOS/TAC), total ammonia nitrogen (TAN), and residual biogas potential (RBP) based on UNI EN ISO 11734:2004 [25] were measured for digestate only, to assess the stability of the anaerobic digestion process. Concerning the evaluation of composting process, total Kjeldahl nitrogen (TKN), phosphorous (P), and potassium (K), were measured on mature compost. Samples for metagenomic analyses were preserved in sterile tubes (50 mL; Sarstedt, Nurnbrecht, Germany) and stored at -20 °C, until use.

### DNA extraction and sequencing

Total DNA was extracted from 200 mg of each sample using the Fast DNA™ SPIN Kit for Soil (MP Biomedicals, LLC, Solon, OH) according to the manufacturer’s protocol. DNA concentration was measured by the Quant-iT dsDNA HS assay kit and the Qubit fluorometer (Invitrogen, Carlsbad, CA, USA), whereas DNA quality was evaluated as previously described [26]. The DNA from the 18 samples was then sequenced at Fasteris (Geneve, Switzerland) using Illumina NovaSeq 6000 technology (Illumina Inc., San Diego, CA), with 2 × 150 paired-end sequencing, producing ~12 Gb of sequences per sample.

### Bioinformatic analyses for MAGs extraction and resistome profiling

The raw reads were quality-assessed and trimmed by adapters using the “Read_qc” module included in the MetaWRAP(v. 0.7) pipeline [27]. Resulting clean reads were assembled using MegaHIT (v. 1.1.3) [28] and the assembled contigs were split into bins using MetaBAT2 (v. 2.12.1) [29] to identify MAGs representative of individual microorganisms. MAGs that were re-evaluated and improved using the “Reassemble_bins” module of MetaWRAP, were then selected for further analyses (Table S2). The quality of the MAGs was assessed using CheckM (v. 1.0.12), focusing on the completeness and contamination metrics [30], whereas the taxonomic classification of MAGs was refined using GTDB-Tk (v. 2.4.0) [31]. The abundance of MAGs (expressed as ‘genome copies per million reads’) among the samples was quantified using the “Quant_bins” module of MetaWRAP. Functional annotation of MAGs for antibiotic resistance genes (ARGs) was performed with Abricate (v. 1.0.0) [32], using three databases: CARD (updated at 2024-Nov-29) [33], NCBI-AMRFinderPlus (updated at 2023-Nov-4) [34] and ResFinder (updated at 2024-Nov-29) [35].

### Extraction and untargeted metabolomic analysis of antibiotic compounds

The extraction step was performed using an ultrasound-assisted extraction (UAE) system (DU-32 ARGOLab, Milan, Italy; maximum power 120 W). Samples (1 g or 1 mL) were ten-fold diluted with an extraction solution consisting in aqueous methanol 80%, acidified with 0.1% formic acid. After the ultrasonic treatment, the extracts were filtered using regenerated cellulose syringe filters (0.22 μm) and collected in amber glass vials until the further analysis. The putative annotation of different antibiotic compounds was done through an untargeted metabolomics approach, based on high-resolution mass spectrometry (HRMS) performed on a Q-Exactive™ Focus Hybrid Quadrupole-Orbitrap Mass Spectrometer (Thermo Scientific, Waltham, MA, USA) coupled to a Vanquish ultra-high-pressure liquid chromatography (UHPLC) pump and equipped with heated electrospray ionization (HESI)-II probe (Thermo Scientific, USA). Chromatography was based on the utilization of water and acetonitrile as mobile phases, both acidified with 0.1% formic acid, considering a gradient elution (6–94% acetonitrile in 35 min). The separation was achieved on a Waters BEH C18 column (2.1 × 100 mm, 1.7 μm). The HRMS conditions were adapted from previously published works [36]. Particularly, the following conditions were used: 200 µL/min as flow rate, a full scan MS analysis (80–1200 m/z) with a mass resolution of 70,000 at m/z 200, a positive heated electrospray ionization system (HESI), and an injection volume equal to 6 µL. The automatic gain control target and the maximum injection time parameters are reported elsewhere [36]. To reach level 2 of confidence in annotation, randomized injections of a pooled quality control (QC) sample were acquired in a data-dependent (Top *N* = 3) MS/MS mode with full scan mass resolution reduced to 17,500 at m/z 200. The Top N ions were selected for fragmentation under stepped (10, 20, 40 eV) Normalized Collisional Energy. Also, the pooled QC sample allowed for the evaluation of method efficiency and reproducibility across the analytical batch. The HESI parameters for both MS and MS/MS were as follows: sheath gas flow 40 arb (arbitrary units), auxiliary gas flow 20 arb, spray voltage 3.5 kV, capillary temperature 320 °C. Finally, the mass spectrometer was calibrated using Pierce™ positive ion calibration solution (Thermo Fisher Scientific, San Jose CA, USA). Raw LC-HRMS data (.RAW files) were processed using MS-DIAL (version 4.90) [37] for automatic peak detection, deconvolution, alignment, and annotation. LOWESS normalization was applied to correct for signal drift and batch effects. The identification of antibiotic compounds was performed via spectral matching against the MoNA (MassBank of North America) public spectral library and a custom antibiotic database compiled from Jonkers et al. [38]. The MS and MS/MS tolerances were set at 0.05 and 0.1 Da, respectively. Retention time was not considered in the total identification score, which had to exceed 50% for a compound to be retained. Relative abundances of the annotated antibiotic compounds were determined based on the normalized peak areas obtained after LOWESS correction. These values, expressed in arbitrary units, reflect the semi-quantitative abundance of each antibiotic across samples and were used in all subsequent statistical analyses and data visualizations. This untargeted, semi-quantitative approach is consistent with methodologies previously described in Jonkers et al. [38] and allows for the comparison of relative signal intensities between experimental groups.

### Multivariate statistical analyses

The metagenomic data were statistically analyzed and visualized using the vegan [39] and ggplot2 [40] packages included in R (v. 4.4.1), to calculate alpha-diversity based on the Shannon index, and beta-diversity using Principal Coordinates Analysis (PCoA) based on Bray-Curtis dissimilarity. False discovery rate (FDR) < 0.05 and fold-change value log2(FC) > 2 were used to determine significant ARGs differences between samples. The metabolomic dataset, after data normalization, underwent principal component analysis (PCA) and hierarchical clustering based on Ward and Euclidean metrics, by using MetaboAnalyst (v.6.0). Statistical significance was assessed using the same thresholds for the metagenomic dataset (FDR < 0.05 and log2(FC) > 2).

Omics integration was performed using Multi-Omics Factor Analysis (MOFA) [41] implemented through OmicsAnalyst (v2.0) [42]. Pearson’s correlation analysis was carried out on the MOFA selected features and considering a Pearson coefficient > 0.70, to investigate the relationship between the two datasets (i.e., ARGs and antibiotic compounds).

## Results

### Biowaste treatment plant performance

The plant handled up to 80,000 t/year of incoming waste, divided into 60,000 t/year of OFMSW, destined for the anaerobic digestion line, and 20,000 t/year of compostable waste (mainly municipal green waste). The outflows from the plant are therefore made up of biomethane (~ 5,500,000 Sm^3^ /year) and mature compost (~ 7,000 t/year). The monitored biochemical parameters from digestate samples indicated a stable anaerobic digestion process. Indeed, pH, FOS/TAC, and TAN values showed levels of 7.90 ± 0.10, 0.190 ± 0.01, and 1,602 ± 305 mg N-NH_4_^+^/kg, respectively, while a RBP of 101.2 ± 11.1 Nm^3^ CH_4_/t_VS_ was found in digestate. Regarding the biochemical parameters monitored to evaluate the composting process, mature compost showed a TKN, P, and K profiles of 12,363 ± 2,189, 6,867 ± 599, and 7,381 ± 1,906 mg/kg, respectively.

### MAGs diversity and distribution

**Fig. 2:**
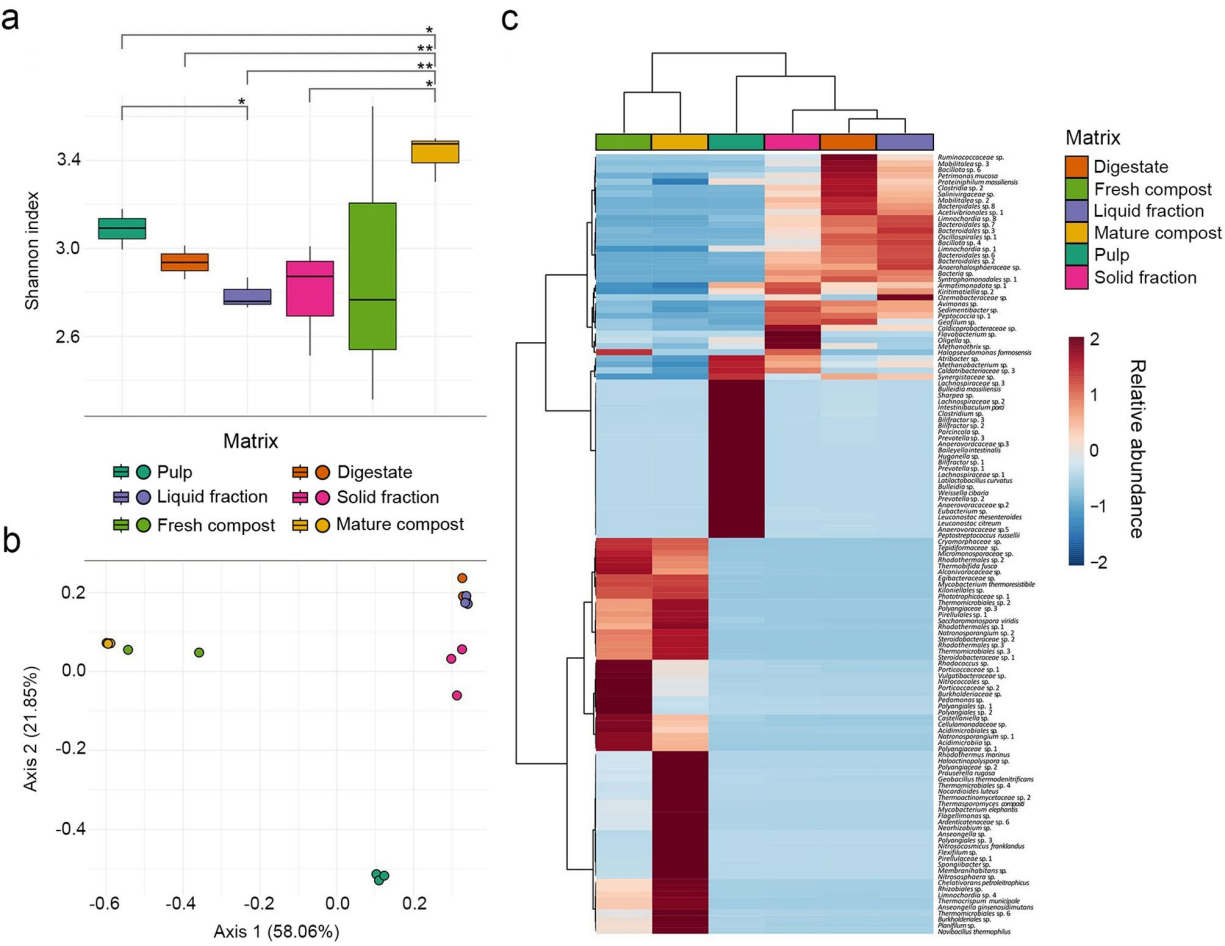
(**a**) Shannon alpha-diversity index of MAGs across different matrices (* = *p*-value < 0.05, ** = *p*-value < 0.01). (**b**) beta-diversity analysis using PCoA based on the Bray-Curtis index for MAGs. (**c**) Hierarchical clustering (Euclidean-based) heatmap of 128 MAGs with a relative abundance ≥ 1% in at least one sample, considering all six matrices

Considering microbial samples diversity, alpha-diversity analysis based on Shannon index (Fig. 2a) showed that all sample categories except fresh compost had a significant (*p*-value < 0.05) lower microbial richness and evenness than mature compost, which showed the highest diversity index. It is worth noting that fresh compost showed the highest variability. Considering beta-diversity (Fig. 2b), the PCoA explained, on the two axes, 79.9% of the overall samples’ diversity, showing a significant difference (*p*-value = 3.2E-11) between the microbial communities of the investigated sample categories. Indeed, a clear clustering was observed among pulp, AD samples (digestate, solid and liquid fractions), and AC samples (fresh and mature compost).

Based on taxonomic classification, 128 MAGs were identified, showing varying relative abundance across sample categories (Fig. 2c). Distinct microbial consortia were associated with each sample type. Pulp samples harboured 26 MAGs, including *Weissella cibaria*, *Leuconostoc mesenteroides*, *Leuconostoc citreum*, *Intestinibaculum porci*, *Bulleidia massiliensis*, *Baileyella intestinalis*, and *Peptostreptococcus russellii*.

Anaerobic digestion (AD) samples—including digestate, liquid, and solid fractions—shared several bacterial MAGs but also exhibited sample-specific microbiota. Digestate was enriched in 10 MAGs, including *Petrimonas mucosa* and *Proteiniphilium massiliensis*. The liquid fraction was dominated by 10 MAGs, particularly four *Bacteroidales* spp., two *Limnochordia* spp., and *Ozemobacteraceae* sp.; while the solid fraction contained 14 abundant MAGs, including *Halopseudomonas formosensis*, *Caldicoprobacteraceae* sp., *Flavobacterium* sp., and *Oligella* sp.

Compost samples were mainly characterized by 65 MAGs. In fresh compost, *Rhodococcus* sp., two *Porticoccaceae* spp., two *Polyangiales* spp., *Vulgatibacteraceae* sp., and *Burkholderiaceae* sp. were dominant. In contrast, mature compost was enriched in *Rhodothermus marinus*, *Mycobacterium elephantis*, *Mycobacterium thermoresistibile*, *Geobacillus thermodenitrificans*, *Thermasporomyces composti*, *Prauserella rugosa*,* Nocardioides luteus*,* Thermocrispum municipale*, *Chelativorans petroleitrophicus*, and *Novibacillus thermophilus*.

Among archaeal MAGs, *Methanothrix* sp. was specific to the solid fraction, while *Methanobacterium* sp. was more abundant in the solid fraction and pulp compared to the liquid fraction and digestate. *Nitrososphaera* sp. and *Nitrosocosmicus franklandus* were predominant in mature compost.

### Resistome composition and distribution

**Fig. 3:**
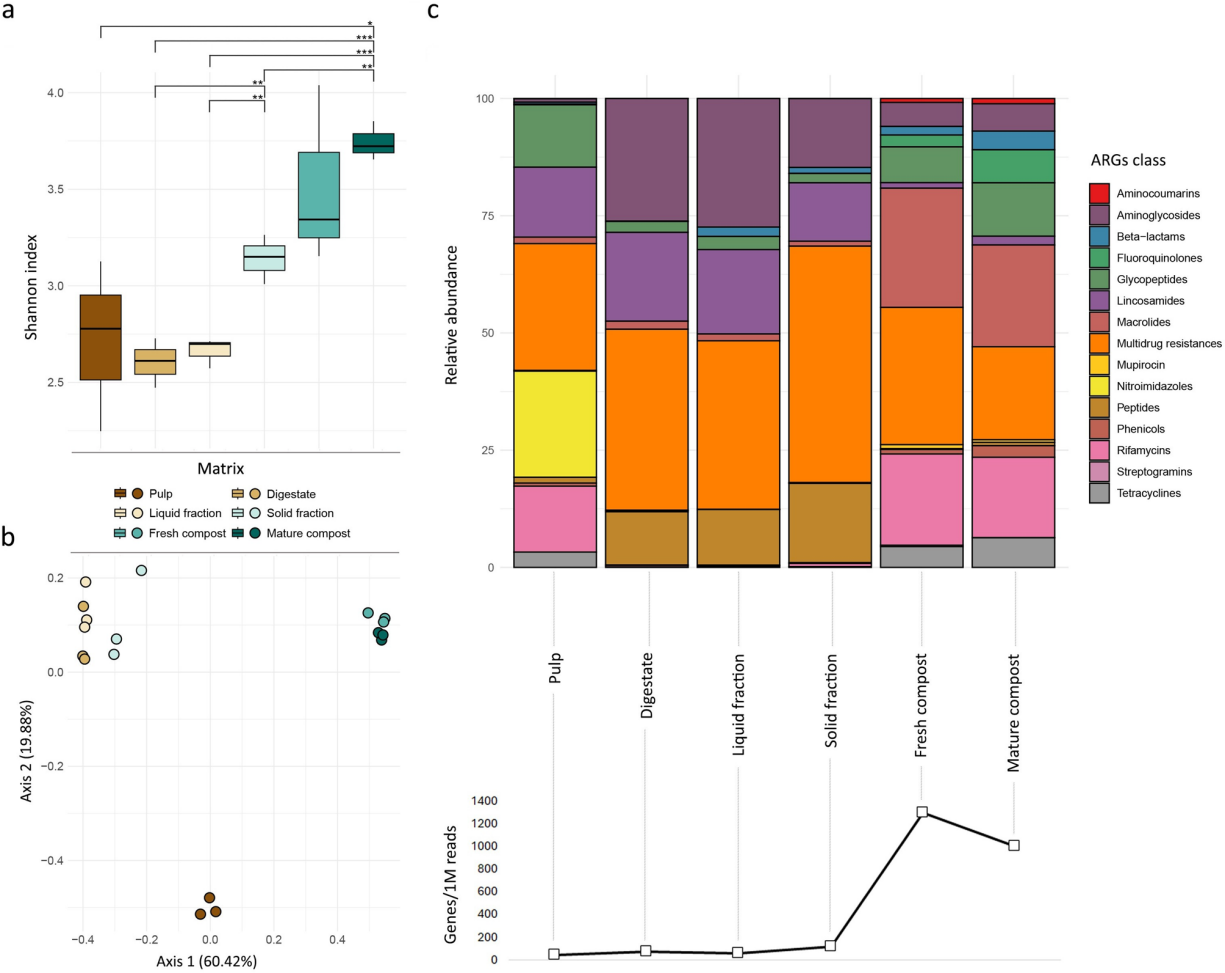
(**a**) Shannon alpha-diversity index of ARGs across different matrices (* = *p*-value < 0.05, ** = *p*-value < 0.01, *** = *p*-value < 0.001). (**b**) Beta-diversity analysis using PCoA based on the Bray-Curtis index for ARGs. (**c**) Average relative abundance of ARG classes, considering ARGs with ≥ 1% abundance in each sample matrix, including the cumulative abundance per matrix expressed as genes per 1 M reads

Considering the ARGs content among matrices, alpha-diversity analysis based on Shannon index (Fig. 3a) showed that pulp (H = 2.7 ± 0.4), digestate (H = 2.6 ± 0.1) and liquid fraction (H = 2.7 ± 0.1) had a significant (*p*-value < 0.05) lower richness and evenness than solid fraction (H = 3.1 ± 0.1), fresh (H = 3.5 ± 0.5) and mature compost (H = 3.7 ± 0.1). On the other hand, pulp and fresh compost had the highest variability. Regarding the beta-diversity, PCoA explained, on the two axes, the 80.3% of the overall samples’ diversity. A significative difference (*p*-value = 5E-15) in the resistome was found among pulp, AD samples (digestate, solid and liquid fractions), and AC samples (fresh and mature compost) (Fig. 3b). Considering the number of ARGs/1 M reads (Fig. 3c), it could be noticed that during the anaerobic digestion process no differences in ARGs abundance were detected from the incoming pulp to digestate and its fractions. In contrast there was a significative increase in ARGs content in the aerobic composting phase, both in fresh (FDR = 0.0008; log2(FC) = 4.1) and mature (FDR = 0.0033; log2(FC) = 3.7) compost. The resistome included 15 drug classes differently distributed among the six matrices (Fig. 3c). Multidrug resistances, nitroimidazoles, lincosamides, rifamycins and glycopeptides ARGs classes mostly represented the pulp samples. In addition to multidrug resistances and lincosamides, aminoglycosides and peptides were dominant in digestate, solid and liquid fractions, whereas in compost samples (both fresh and mature), macrolides, rifamycins, tetracyclines, fluoroquinolones, and glycopeptides, were the most prevalent.

To evaluate significant differences in ARGs classes between the main steps of the considered biological treatments (i.e., anaerobic digestion, digestate fraction separation and aerobic composting), a FC analysis combined with a t-test was performed. Digestate and mature compost significantly differed for 13 drug classes out of the 15 previously described (Table 1). Specifically, considering the FC values, 9 ARGs classes were enriched in mature compost, with the highest values for fluoroquinolones, aminocoumarins and streptogramins classes. Instead, 4 ARGs classes were enriched in digestate, with the highest value for nitroimidazoles. Between pulp and digestate, 5 ARGs classes out of 15 showed a different pattern of enrichment: rifamycins, streptogramins and aminocoumarins for pulp, and aminoglycosides and peptides for digestate. Regarding the two comparisons, liquid vs. solid fraction, and fresh vs. mature compost, no significant differences were evidenced.

**Table 1:** Statistical analysis of the distribution of relative abundances of ARG classes across sample matrices based on FC analysis and t-test (UP: accumulated ARG class in digestate; DOWN: accumulated ARG class in mature compost/pulp)

Digestate (UP) vs. Mature Compost (DOWN)		
ARGs class	**log2(FC)**	**p.adjusted**
Rifamycin	-5.5534	3.24E-04
Macrolide	-3.6574	6.48E-04
Beta-lactamase	-6.2075	9.35E-04
Lincosamide	3.3385	0.0017921
Aminoglycoside	2.1653	0.0022505
Tetracycline	-6.4804	0.016248
Peptide	3.9975	0.016248
Mupirocin	-3.9123	0.016248
Streptogramin	-7.2862	0.016785
Aminocoumarin	-9.3206	0.016855
Nitroimidazole	9.9696	0.018177
Fluoroquinolone	-14.717	0.021194
Glycopeptide	-2.2889	0.039047
***Digestate (UP) vs. Pulp (DOWN)***		
**ARGs class**	**log2(FC)**	**p.adjusted**
Aminoglycoside	5.1877	0.0038589
Rifamycin	-5.2663	0.012109
Streptogramin	-4.8315	0.038349
Peptide	3.2138	0.038349
Aminocoumarin	-5.3541	0.047816

**Fig. 4:**
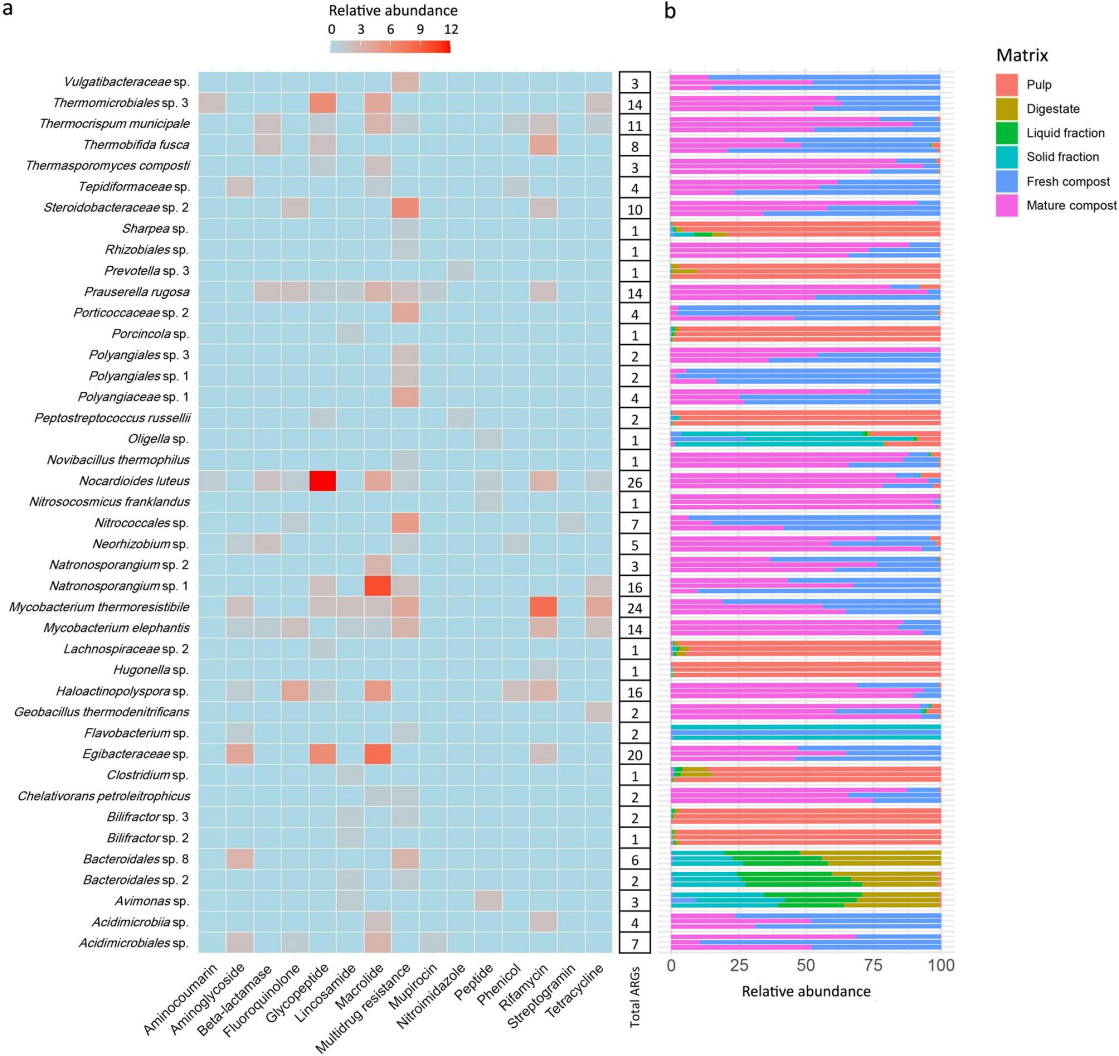
(**a**) Frequency of ARGs grouped by drug class for each MAG. (**b**) Relative abundance of MAGs across the six matrices

MAGs harbouring the highest number of ARGs (≥ 10) were (Fig. 4a, Table S3): *Nocardioides luteus*, mostly including glycopeptide (*van*), macrolide (*ole*C, *mgt*) and rifamycin (*rgt1438*, *arr-1*) genes. *Mycobacterium thermoresistibile* and *Mycobacterium elephantis* consisting mainly of multidrug resistance (*sox*R, *efp*A), rifamycin (*rox*, *arr-4*, *arr-*ms, *rpo*B, *rpo*B2), and tetracycline (*tet*V) genes. *Thermomicrobiales* sp.3 mostly showing glycopeptide (*van*) and macrolide (*ole*C) genes. *Thermocrispum municipale* mostly including macrolide genes (*ole*C), *Steroidobacteraceae* sp.2 multidrug resistance genes (*rsm*A, *sme*D, *smeE*), *Haloactinopolyspora* sp. fluoroquinolone (*qep*A1, *qep*A2, *qep*A4), macrolide (*ole*C) and rifamycin (*rox*) genes, *Egibacteraceae* sp. aminoglycoside (*aac(6’)-Isa*, *aac(6’)-Ib-sk*), glycopeptide (*van*) and macrolide (*ole*C) genes. *Natronosporangium* sp.1 mostly including macrolide genes (*ole*C), whereas *Prauserella rugosa* mostly showing beta-lactam (*bla*), multidrug resistance (*erm*), macrolide (*ole*C, *mph*H), fluoroquinolone (*qep*A), and rifamycin (*rph*) genes. These MAGs were mainly present in mature compost, followed by fresh compost (Fig. 4b).

Considering MAGs harbouring a number of ARGs < 10, there were (Fig. 4a, Table S3): *Thermobifida fusca* mostly including rifamycin genes (*rox*, *rpo*B, *rpo*B2), *Polyangiaceae* sp. 1 and *Nitrococcales* sp. multidrug resistance genes (*mph*A, *opr*A, *opr*Z, *mex*B, *mex*C, *sme*D, *tmex*D4, *sox*R, *axy*Y), *Acidimicrobiia* sp. macrolide (*ole*C) and rifamycin (*rpo*B) genes, *Acidimicrobiales* sp. aminoglycoside (*aac*A*-str-10*, *aac(6’)-Isa*) and macrolide (*ole*C) genes. These MAGs were also mainly present in compost samples, particularly in fresh compost (Fig. 4b). Instead, *Prevotella* sp.3 and *Peptostreptococcus russelii* included ARGs belonging to nitroimidazole class (*nim*J, *nim*B) and they were mainly present in pulp. Finally, it was noticed that *Bacteroidales* spp. and *Avimonas* sp. mostly included aminoglycoside (*ran*A), lincosamide (*lnu*H), multidrug resistance (*erm*N, *Isa*E) and peptide (*pmr*E) genes. These MAGs were specifically found in digestate and its fractions, both liquid and solid (Fig. 4b).

### Identification of antibiotic compounds

**Fig. 5:**
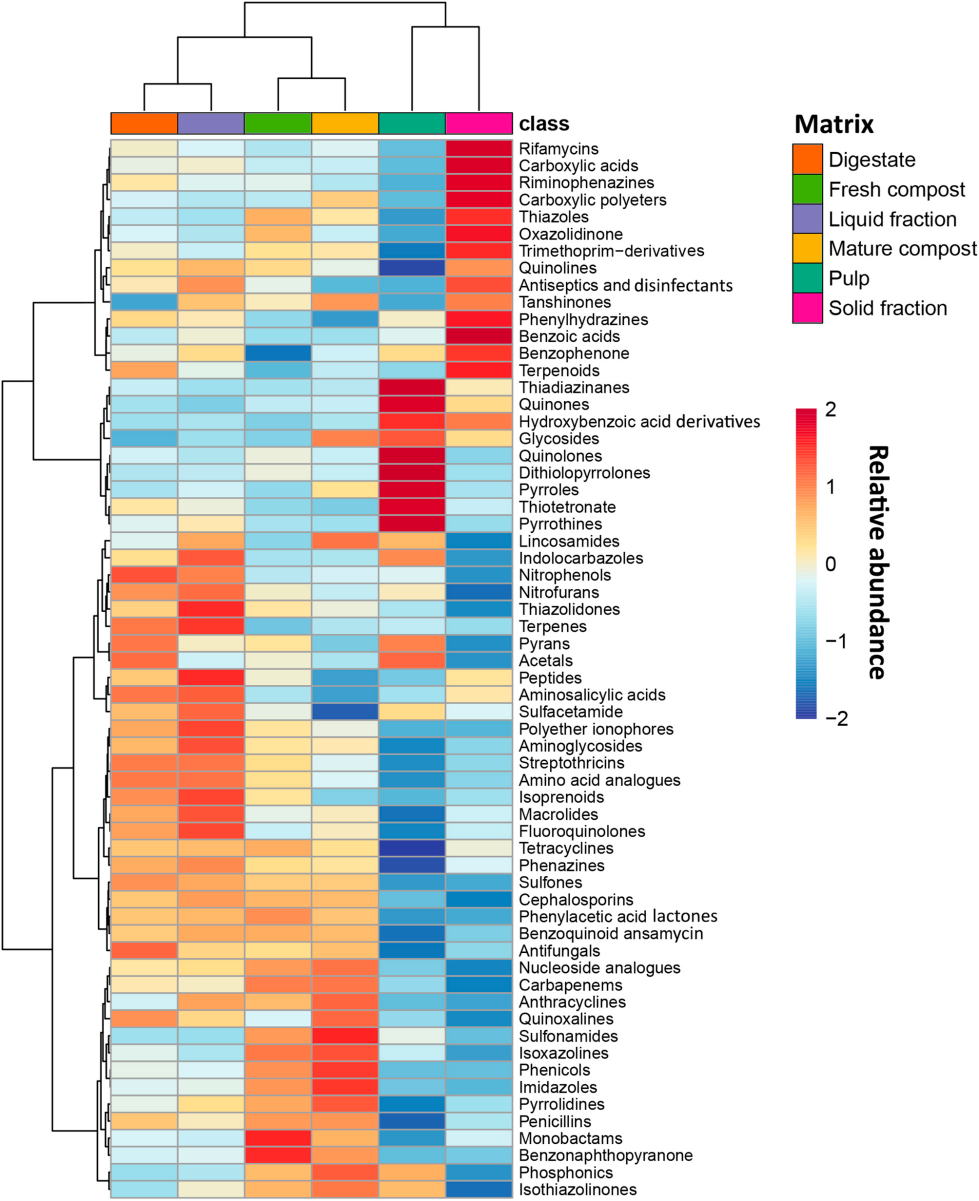
Hierarchical clustering (Euclidean-based) of antibiotic drug classes distributed across the six matrices

The annotated compounds found are reported in Table S4, considering their MS1-isotopic profile, identification score, and relative abundance values. As the first step, all the 354 identified antibiotics were grouped into their main chemical classes, thus extrapolating a cumulative abundance value for a total of 62 classes of compounds. Thereafter, these main classes were used to perform unsupervised statistical approaches, namely PCA and hierarchical clustering. The PCA model (Figure S1) explained a cumulative 71.9% of the total variability across the first three principal components. A clear clustering was observed among pulp, AD samples (digestate, solid and liquid fractions), and AC samples (fresh and mature compost). Notably, within the AD group, the solid fraction exhibited a distinct antibiotic profile compared to both the digestate and the liquid fraction. Additionally, the heatmap shown in Fig. 5 revealed a differential and exclusive pattern of enrichment of selected antibiotic classes that clearly characterized the different sample categories. Particularly, two main clusters of samples were outlined: on one side digestate, liquid fraction, fresh compost, and mature compost were found, while on the other side a more exclusive antibiotic profile was shown for pulp and solid fractions. Looking at the most significative drug classes, it was noted that each matrix was characterized by specific cores (i.e., up- vs. down- enriched classes of compounds). Indeed, the comparison between digestate and pulp (Table 2), as indicated by fold-change (FC) analysis, demonstrated a significant enrichment of hydroxybenzoic acid derivatives and dithiolopyrrolones in pulp, whereas thiazoles, phenazines, and trimethoprim derivatives were predominantly present in digestate. The analysis of the AD fractions (liquid vs. solid) revealed a significant increase of rifamycins and thiazoles relative abundance in the solid fraction, while indolocarbazoles and thiazolidones were more prevalent in the liquid fraction. Furthermore, when comparing digestate to mature compost, digestate exhibited a significant increase of phenylhydrazines and aminosalicylic acids relative abundance, whereas mature compost was characterized by the presence of imidazoles, isoxazolines, sulfonamides, and phenicols. Finally, the comparison between fresh and mature compost did not reveal any significant differences.

**Table 2:** Statistical analysis of the distribution of relative abundances of antibiotic drug classes across sample matrices based on FC analysis and t-test (UP: accumulated drug class in digestate/liquid fraction; DOWN: accumulated drug class in mature compost/pulp/solid fraction)

Digestate (UP) vs. Mature Compost (DOWN)		
**Drug class**	**log2(FC)**	**p.adjusted**
Phenicols	-4.0338	0.0053299
Sulfonamides	-3.134	0.022825
Amino acid analogues	1.7736	0.022825
Isoxazolines	-2.9283	0.02338
Aminosalicylic acids	3.9357	0.024539
Imidazoles	-2.8829	0.024539
Phenylhydrazines	6.4175	0.027365
Phosphonics	-3.1509	0.027365
Streptothricins	1.6771	0.027365
Nucleoside analogues	-1.8043	0.035919
***Digestate (UP) vs. Pulp (DOWN)***		
**Drug class**	**log2(FC)**	**p.adjusted**
Hydroxybenzoic acid derivatives	-8.5026	1.48E-05
Amino acid analogues	3.6125	5.75E-04
Macrolides	2.8026	5.75E-04
Pyrrothines	-4.1001	7.79E-04
Phenazines	4.2653	0.0023707
Aminosalicylic acids	3.0546	0.0023707
Antifungals	2.8603	0.0023707
Tetracyclines	3.1536	0.0028772
Sulfones	2.9268	0.0063554
Nucleoside analogues	2.021	0.0065191
Dithiolopyrrolones	-7.8409	0.0066331
Aminoglycosides	2.7737	0.0066331
Phosphonics	-2.011	0.0088441
Oxazolidinone	2.5656	0.0089468
Cephalosporins	1.5618	0.0089468
Penicillins	2.4968	0.010168
Quinones	-7.5907	0.010781
Streptothricins	3.3865	0.010781
Phenicols	2.2222	0.010781
Thiadiazinanes	-6.3059	0.011688
Fluoroquinolones	1.4183	0.011688
Thiotetronate	-2.4887	0.012676
Quinolones	-2.3438	0.016958
Quinolines	1.8146	0.017247
Glycosides	-2.7739	0.017768
Trimethoprim-derivatives	4.005	0.026663
Peptides	1.7879	0.033123
Isothiazolinones	-1.063	0.034545
Nitrophenols	2.4796	0.035346
Isoprenoids	3.241	0.037338
Quinoxalines	2.3753	0.039763
Thiazoles	4.6381	0.041852
Polyether ionophores	2.9222	0.043938
***Liquid fraction (UP) vs. Solid fraction (DOWN)***		
**Drug class**	**log2(FC)**	**p.adjusted**
Riminophenazines	-6.075	8.14E-04
Carboxylic acids	-5.6132	8.95E-04
Carboxylic polyeters	-8.0465	0.0018883
Phenylhydrazines	-6.8831	0.0018883
Hydroxybenzoic acid derivatives	-6.6116	0.0018883
Indolocarbazoles	4.6384	0.0018883
Thiazolidones	3.7066	0.0018883
Rifamycins	-8.5942	0.0031475
Trimethoprim-derivatives	-4.5727	0.0036086
Cephalosporins	2.2902	0.0091484
Nucleoside analogues	3.2659	0.0099492
Macrolides	1.7956	0.0099492
Benzoic acids	-6.7291	0.012724
Thiadiazinanes	-2.2856	0.012844
Benzophenone	-3.2162	0.013751
Aminoglycosides	2.4491	0.018296
Quinones	-4.1569	0.018547
Aminosalicylic acids	1.9438	0.018547
Thiazoles	-9.9309	0.019432
Amino acid analogues	2.5733	0.019432
Terpenoids	-6.632	0.022153
Nitrofurans	2.4991	0.023464
Polyether ionophores	3.2012	0.024629
Phenicols	1.7868	0.034472
Isothiazolinones	1.5952	0.036822
Pyrrothines	1.5671	0.038162
Sulfacetamide	3.7847	0.049154
Phenylacetic acid lactones	1.9301	0.049154
Imidazoles	1.6251	0.049154

**Fig. 6:**
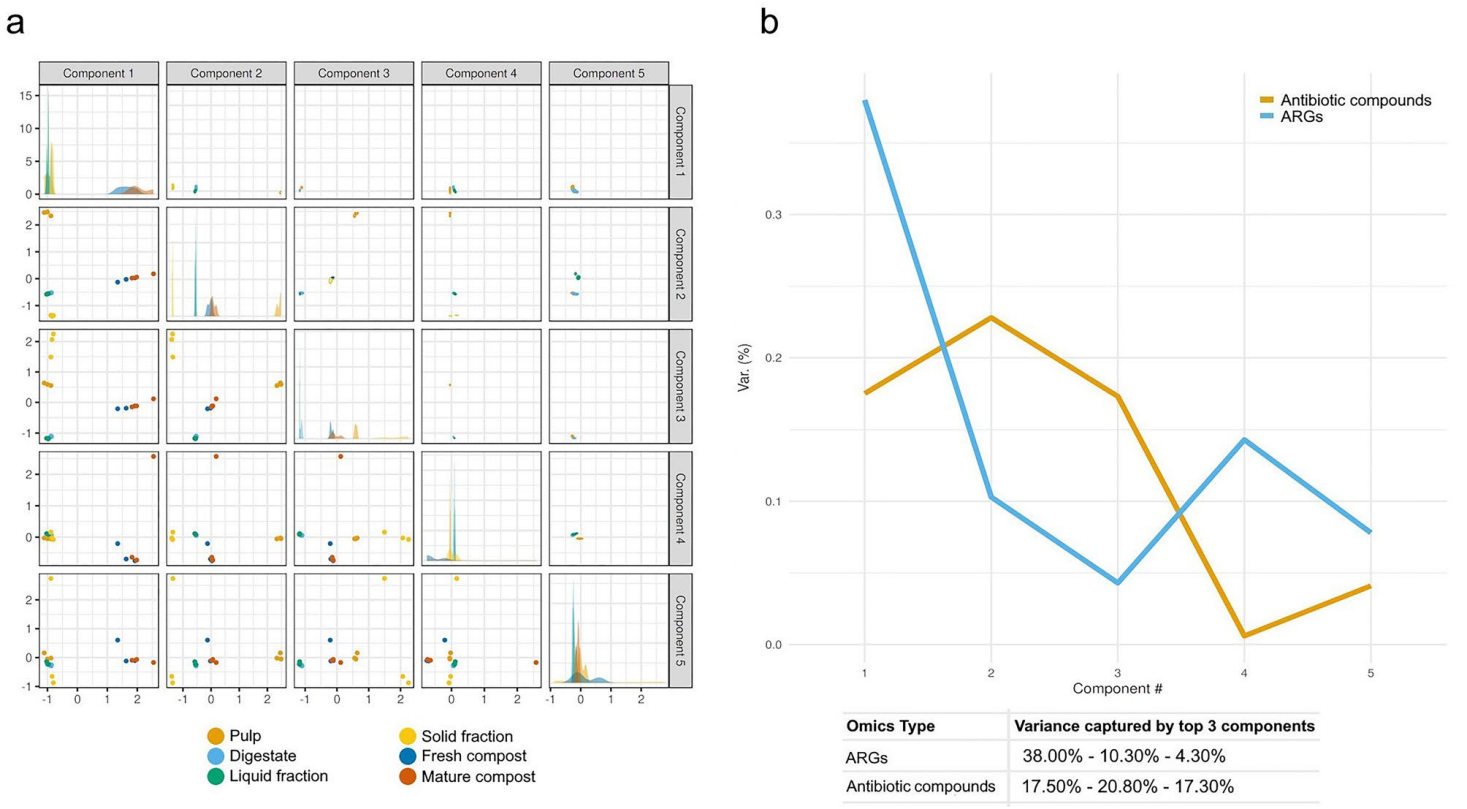
(**a**) Score plot showing the distribution of samples across the first five components identified by multi-omics factor analysis (MOFA); (**b**) Line graph representing the first five components of the MOFA model, showing the percentage of variance explained across the two -omics datasets

To explore putative relationships between antibiotic compound profiles (i.e., untargeted metabolomics dataset) and antibiotic resistance genes (i.e., metagenomics dataset), an unsupervised integrative framework (i.e., MOFA analysis) was applied to discover latent factors that capture shared sources of variability across different -omic levels. As shown in Fig. 6a, MOFA revealed a clear separation among sample categories based on the first five components. Notably, fresh and mature compost samples clustered distinctly from the other matrices (pulp, digestate, liquid and solid fractions), especially along Component 1 and Component 2. Figure 6b depicts the proportion of variance captured by the top five MOFA components for each -omic layer, revealing a partially coordinated structure between the two datasets. Specifically, component 1 accounted for 38.0% of the variance at the resistome level and 17.5% at the metabolomic level, suggesting that this factor may reflect a biological signal that influences both -omic domains. In contrast, components 2 and 3 captured 20.8% and 17.3% of the metabolomic variance, respectively, but explained only 10.3% and 4.3% of the variance in the resistome, indicating more specific sources of variation in the dataset. To refine the analysis and focus on the most informative features, the variables that contribute most to each -omic level were selected based on MOFA loadings and subjected to Pearson correlation analysis (Table S5). Glycopeptide and multidrug-resistance ARG classes showed the highest number of positively correlated antibiotic compounds (i.e., 30.7% and 20.6%, respectively). In addition, the β-lactamase ARG class showed the highest number of positive correlations with compounds belonging to the β-lactam antibiotic class. Aminoglycoside, fluoroquinolone, and macrolide ARG classes also showed positive correlations, each associated with 1 to 3 compounds from the corresponding antibiotic class. In contrast, no positive correlations were observed between the remaining ARG classes and compounds in the same class.

## Discussion

The samples analysed in this study were collected from two biological integrated processes, anaerobic digestion and aerobic composting, operating under steady-state conditions. These conditions were confirmed by a constant monitoring of the main physico-chemical parameters (e.g., pH, FOS/TAC and TAN) [43]. Moreover, RBP values of the analysed digestate samples suggested a well-operating AD plant since an efficient and biologically stable anaerobic digestion usually produces digestate with RBP values ranging between 85 and 165 Nm³CH_4_/t_VS_ [44, 45]. Considering the analyses of TKN, P and K performed on the mature compost, the results found also confirm the achievement of common chemical characteristics at the end of aerobic composting, due to the microorganisms present at the latter stage, that lead to degradation of organic matter and to the release of the above elements [46].

### Core MAGs shaped by the different steps involved in the OFMSW integrated treatment

The processes of anaerobic digestion and aerobic composting led to a sharp differentiation of the microbial communities present in the pulp, mostly characterized by species belonging to the *Bacillota* phylum and related to the production of acetate, lactate, and ethanol during carbohydrate and organic waste fermentation [47]. Few species, such as *Methanobacterium* sp. and *Synergistaceae* sp., that are generally considered crucial in the AD process, were also evidenced in addition to the AD samples, since the liquid fraction of digestate was recirculated to homogenize the incoming organic waste and constitute the pulp. The AD process highly selected the microbial composition, including species with a pivotal role in the hydrolysis phase of anaerobic digestion (e.g., *Ruminococcaceae* sp., *Clostridia* sp., *Limnochordia* sp., *Bacteroidales*,* Petrimonas mucosa* and *Proteiniphilum massiliensis*), as they are able to ferment carbohydrates (as well as other compounds such as proteins or amino acids) from organic sources like OFMSW [48–52]. The conversion of these organic compounds in CO_2_ and acetate is synergic with the production of methane at the later stage of AD [53, 54]. The selection of species involved in protein degradation and reduction of nitrate to nitrite under anaerobic conditions (e.g., *Oligella* sp., *Flavobacterium* sp.) [55] was also highlighted in AD samples. The accumulation of nitrogen, resulting from the hydrolysis of protein-rich substrates such as OFMSW, indeed could lead to a subsequent accumulation of ammonium, which negatively affects both the overall performance of the reactor and the methanogenic phase [56]. Regarding the latter phase, the methanogenic community was mostly represented by the acetoclastic *Methanotrix* sp [57].

The composting process strongly increased the microbial diversity, also altering its taxonomic composition. This observation is consistent with previous studies reporting that composting, when applied after anaerobic digestion (AD), enhances both taxonomic richness and community complexity. While AD supports a narrower set of anaerobic microorganisms, the transition to aerobic composting conditions introduces new ecological niches—due to changes in oxygen availability, temperature, and substrate composition—that promote the proliferation of diverse microbial taxa. Several authors have documented increases in microbial richness and evenness during composting of digestate, with enrichment in groups such as *Actinobacteria*, *Firmicutes*, *Bacteroidetes*, *Planctomycetes*, and *Verrucomicrobia*, which are associated with the degradation of complex organic matter and the maturation of compost [58–60]. These community shifts reflect microbial succession processes that result in more stable and functionally diverse ecosystems compared to the original digestate [60].

In this study, dominant thermophilic *Actinomycetes* species such as, *Thermasporomyces composti*,* Mycobacterium elephantis*,* Mycobacterium thermoresistibile*, and *Thermocrispum municipale*, along with the mesophilic *Nocardioides luteus*, were highlighted at different step of the process, resulting in fresh and mature compost [61–63]. These species include the ability to degrade a wide range of compounds. For instance, *N. luteus* can degrade aromatic compounds, hydrocarbon and haloalkane pollutants, nitrogen-containing heterocyclic compounds, and polymeric polyester substances [64]. A prevalence of *Geobacillus thermodenitrificans* was also observed in compost samples. This species has been previously highlighted for its H_2_S oxidation role in composting, reducing H_2_S emission and increasing sulfate content [65]. Moreover, a characteristic *Archaea* community was highlighted in compost, including *Nitrososphaera* sp. and *Nitrosocosmicus franklandus*, which are ammonia-oxidizing archaeon (AOA), generally present in soils. These species contribute to the oxidation of NH_3_ in the nitrification process that occurs in soils subjected to fertilization, thus highlighting the potential it could have in the spreading of mature compost [66].

### MAGs community structure drives ARGs diversity and abundance

Within the OFMSW-treating integrated process a significant change in both ARGs diversity and distribution was highlighted between AD and AC samples. Overall, the annotated ARGs were classified into 15 different drug classes, with multidrug resistances, aminoglycosides, lincosamides, macrolides, rifamycins, glycopeptides, and nitroimidazoles as the most abundant. However, besides the multidrug resistance class, AD samples were mostly dominated by aminoglycoside and lincosamide ARGs, whereas AC samples by macrolide and rifamycin ARGs. Despite differences in drug class dominance, the composting process significantly increased both the ARGs diversity and relative abundance. This change can be mainly due to the operating conditions of the AC process (i.e., high temperatures and oxidative stress) that led to the selection of bacteria harbouring a high and varied ARGs content. It has been pointed out the importance of the maturation phase of compost, since a rapid composting was associated to a sharp reduction in ARGs removal efficiency [12]. Indeed, rapid composting limits humus-like components formation that could exert an inhibitory effect on ARGs proliferations. On the contrary, protein-like compounds can promote the growth of host bacteria, indirectly increasing ARGs abundance [12].

In addition to process conditions, shift in microbial community composition, can be influenced by exposure to other environmental stressors [10], such as microplastics [67, 68], metals [69, 70], and antibiotics themselves [71]. Numerous studies have examined the mechanisms driving the enrichment and dissemination of antibiotic resistance genes (ARGs) under various selective pressures [10]. Microbial populations often secrete extracellular polymeric substances (EPS) as a protective strategy to shield the cells [72]. Moreover, these populations may elevate the levels of reactive oxygen species (ROS), triggering the activation of the SOS response to repair DNA damage. This process can lead to the emergence of new resistance genes when subjected to stressors such as antibiotics [72], artificial sweeteners [73], and surfactants [74]. Alterations in cell membrane permeability and structural damage under these selective pressures may further facilitate the horizontal transfer of ARGs through conjugation [72]. Although these mechanisms were not directly investigated in the present study, they represent conceptual models proposed in previous research that may aid in interpreting the observed patterns of ARG dynamics under multifactorial environmental pressures.

Predominance of *Actinomycetes* MAGs in the composting phase can be consistent with the highest frequency of detected ARGs. *Actinomycetes* are known to produce different classes of antibiotics in terms of chemical structure and mechanisms of action, such as glycopeptide antibiotics [75]. The most abundant ARGs were reported to be *vanR* and *vanS* variants found in the *vanO* gene cluster. Flores-Orozco et al. [76] in their studies pointed out how anaerobic digestion treatment did not lead to the reduction of microorganisms carrying vancomycin resistance genes. The *van* genes are generally associated with their biosynthesis, favouring a possible co-regulation between antibiotic production and self-resistance [77].

Most macrolides resistance genes consisted of the *oleC* gene (oleandomycin), whose mechanism of antibiotic resistance is efflux pump that leads to the excretion of chemical compounds, such as antibiotics, outside the bacterial cell [78]. The abundance of this drug class in AC samples could be linked to the different temperatures adopted during the two different processes (60 ± 5 °C in AC and 42 ± 2 °C in AD). In fact, as described by Zhang et al. [79], the mesophilic anaerobic digestion may be ineffective in the reduction of macrolide genes.

Concerning tetracycline genes, several studies reported the significant decrease of this drug class after aerobic composting process [80]. This trend was not observed in our results, where on the contrary an increase of tetracycline ARGs relative abundance (especially *tetM*) in fresh and mature compost was evidenced. This unexpected trend could result from selective pressure due to residual tetracycline or from co-selection mechanisms, such as the presence of heavy metals or other stressors [81]. The spreading of compost rich in tetracycline genes represents an environmental risk, since antibiotic resistant bacteria harbouring tetracycline genes are able, via horizontal gene transfer, to convert bacteria indigenous of agricultural soils in bacteria carrying ARGs [82].

In the context of assessing the risks associated with ARG-carrying microorganisms in compost-derived materials, it is worth highlighting that mature compost was found to harbor thermotolerant microbes as ARG reservoirs, including higher relative abundance of *Mycobacterium thermoresistibile*, a heat-resistant species within a genus that includes known human and animal pathogens [83]. The observed persistence at composting temperatures underscores the value of exploring additional microbial biomarkers for the surveillance of pathogenic bacteria and ARG reservoirs. Indeed, it has been pointed out that current regulatory thresholds for soil improvers are inadequate for the assessment of AMR risks, and the use of targeted PCR diagnostics has been recommended [84]. Consistently, the Food and Agriculture Organization (FAO) also emphasizes the need for national legislative frameworks to support the development and implementation of specific regulations addressing antimicrobial residues and ARGs in soil, sludge, and waste materials [85].

### Untargeted metabolomics mapping of antibiotic compounds

Untargeted metabolomics was conducted to identify class of antibiotics compound in the different sample categories to better understand the correlations between antibiotic compounds and antibiotic resistance genes (ARGs). The pulp, a by-product of waste pretreatment, contains organic raw materials that can retain high concentrations of antibiotics from various sources [86, 87]. After anaerobic digestion, the digestate mainly enriched phenazines and trimethoprim derivatives. The former could be also present due to the digestate microbial consortia; indeed, several phenazine derivatives have been isolated from different Gram-positive and Gram-negative bacteria as well as from archaeal methanogenic species [88]. On the contrary, trimethoprim derivatives might show enrichment due to limited microbial degradation [89].

The different content of antibiotic compounds found in the digestate fractions after centrifugation, can be mainly due to physicochemical properties. Indeed, the solid fraction preferentially retains hydrophobic and structurally complex compounds, including antibiotics and their degradation products, because of their tendency to adsorb onto organic matter. This differential distribution highlights the role of physicochemical properties in determining the fate of antibiotics in anaerobic digestion systems [90].

Regarding mature compost, phenicols, sulfonamides, isoxazolines, and imidazoles were the most enriched. Imidazoles and isoxazolines, commonly used as antiparasitic agents, may enter compost through the disposal of green waste [91]. On the other hand, the enrichment of phenicols in mature compost could be explained by the presence of producing bacteria such as those of the *Actinomycetes* group present in compost [75, 92]. Conversely, sulfonamides exhibit significant environmental persistence in mature compost [93]. It has been previously pointed out a positive correlation between sulfonamides initial concentrations and removal rates following AC treatment, likely due to elevated selective pressure exerted on microbial communities [15, 94]. However, it is worth highlighting that the degradation or removal of antibiotic compounds can occur through a range of concurrent processes—including adsorption onto organic or inorganic matter, hydrolysis, photodegradation, oxidation, and photooxidation—all of which may vary significantly depending on environmental conditions, microbial activity, and the physicochemical properties of the antibiotics themselves [95].

Overall, untargeted metabolomics revealed distinct distribution patterns for specific classes of antibiotics. Indeed, cluster analysis indicated that anaerobic digestion and composting do not fully degrade some antibiotics but rather redistribute them among different fractions. The distinct antibiotic profiles of the pulp and solid fraction, which serve as the initial substrates for anaerobic digestion and composting, respectively, suggest that biowaste pretreatment and digestate solid–liquid separation may represent critical control points to mitigate antibiotic contamination in the early stages of treatment. Indeed, the identification of antibiotic classes in these matrices provides a valuable foundation for optimizing process parameters and designing targeted quantitative monitoring tools.

Regarding -omics integration, MOFA enabled the identification of latent components that capture shared sources of variability across -omic levels. Correlation analysis revealed class-specific positive correlations between β-lactam, aminoglycoside, fluoroquinolone, and macrolide ARGs classes, and the antibiotic compounds of the same class. This result suggested a covariance of the selected drug classes within the same sample matrix in the two -omics datasets. Thus, the shown positive correlation could indicate that the presence of a specific antibiotic compound and its corresponding resistance gene is driven by selective pressure exerted by that compound itself, which may favor the proliferation of resistant microorganisms and thus shape the overall microbial community structure [10]. In contrast, other classes of antibiotic compounds do not show a correlation with their corresponding ARG classes, suggesting that factors beyond microbial resistant community composition may influence the distribution and abundance of these resistance genes. For instance, abiotic environmental conditions, such as pH, temperature and the presence of metal residues, might play a more significant role in shaping the spread and persistence of ARGs [96, 97].

### Limitations and future perspectives

This study provided insights into the dynamics of antibiotic resistance genes (ARGs) and antibiotic compounds in an integrated process of anaerobic digestion and composting. The metagenomic and metabolomic data, based on relative abundances, offered valuable perspectives on shifts in microbial community structure, resistance genes and antibiotic compounds distribution, even though they are not designed for absolute quantification. Shotgun metagenomics enabled a broad overview of microbial and resistome diversity, while the untargeted metabolomics approach captured a wide range of metabolites without the need for predefined targets. To streamline data interpretation, features were grouped into ARG and antibiotic classes, a strategy that enhances clarity at the functional group level, though it may reduce resolution for detecting specific degradation metabolites related to the primary antibiotic. Future studies could enhance the characterization of microbial functional potential through MAGs functional annotation, providing deeper insights into the metabolic processes involved in the integrated anaerobic digestion and composting system. Additionally, considering seasonal variation could provide evidence of temporal shifts in ARG and antibiotic profiles associated with fluctuations in feedstock composition, especially the decline in green waste during winter.

Understanding the fate of antibiotics throughout the various stages of anaerobic digestion and composting at full-scale operation remains particularly challenging due to the complex interplay of multiple biotic and abiotic factors. However, the study identified key trends that can serve as a foundation for future research using complementary quantitative and targeted approaches.

## Conclusions

This study has shown how integrated -omics techniques as metagenomics and untargeted metabolomics can be systematically utilized to detect emerging ARGs and antibiotic compounds dynamics and monitor their ongoing evolution in biological waste treatment plants. The findings highlight that the two treatment processes—anaerobic digestion and composting—select for distinct classes of ARGs and antibiotic compounds, reflecting different microbial and physico-chemical selection pressures. Moreover, in terms of ARG frequency, mature compost was found to harbor a higher abundance, which is associated with a greater relative abundance of resistant bacterial taxa. These insights emphasize the need to tailor treatment strategies to the specific resistome and chemical profiles they promote, and they underline the potential of -omics approaches in informing risk assessment and guiding the safe and sustainable reuse of treated organic waste.

## Supplementary Information

Below is the link to the electronic supplementary material.

Supplementary Material 1

## Data Availability

The data that supports the findings of this study are available in the tables and additional files of this article. Sequences were deposited to the Sequence Read Archive of NCBI under the BioProject PRJNA1225463.

## Abbreviations

OFMSW: Organic fraction of municipal solid waste
AD: Anaerobic digestion
AC: Aerobic composting
ARG: Antibiotic resistance gene
MAG: Metagenome-assembled genome
FDR: False discovery rate
FC: Fold-change
TS: Total solid
VS: Volatile solid
TAN: Total ammonia nitrogen
TKN: Total Kjeldahl nitrogen
RBP: Residual biogas potential
FOS/TAC: Acidity/alkalinity ratio
H: Shannon index
MOFA: Multi-omics factor analysis
